# Vaccine Development to Treat Alzheimer's Disease Neuropathology in APP/PS1 Transgenic Mice

**DOI:** 10.1155/2012/376138

**Published:** 2012-09-16

**Authors:** Iván Carrera, Ignacio Etcheverría, Lucía Fernández-Novoa, Valter Lombardi, Ramón Cacabelos, Carmen Vigo

**Affiliations:** ^1^Department of Neurosciences, EuroEspes Biotechnology, Polígono de Bergondo, Nave F, 15165 A Coruña, Spain; ^2^EuroEspes Biomedical Research Center, Institute for CNS Disorders and Genomic Medicine and EuroEspes Foundation, 15166 La Coruña, Spain; ^3^Neuroscience Division, Atlas Pharmaceuticals, Sunnyvale, CA 94089, USA

## Abstract

A novel vaccine addressing the major hallmarks of Alzheimer's disease (AD), senile plaque-like deposits of amyloid beta-protein (A**β**), neurofibrillary tangle-like structures, and glial proinflammatory cytokines, has been developed. The present vaccine takes a new approach to circumvent failures of previous ones tested in mice and humans, including the Elan-Wyeth vaccine (AN1792), which caused massive T-cell activation, resulting in a meningoencephalitis-like reaction. The EB101 vaccine consists of A*β*_1-42_ delivered in a novel immunogen-adjuvant composed of liposomes-containing sphingosine-1-phosphate (S1P). EB101 was administered to APPswe/PS1dE9 transgenic mice before and after AD-like pathological symptoms were detectable. Treatment with EB101 results in a marked reduction of A**β** plaque burden, decrease of neurofibrillary tangle-like structure density, and attenuation of astrocytosis. In this transgenic mouse model, EB101 reduces the basal immunological interaction between the T cells and immune activation markers in the affected hippocampal/cortical areas, consistent with decreased amyloidosis-induced inflammation. Therefore, immunization with EB101 prevents and reverses AD-like neuropathology in a significant manner by halting disease progression without developing behavioral spatial deficits in transgenic mice.

## 1. Introduction

Alzheimer's disease (AD) is the most frequent form of dementia in developed countries, with a prevalence of about 1% at the age of 65 and over 25% in people older than 85 years of age. Clinically, it is characterized by progressive cognitive deterioration, behavioral disturbances, and functional decline. It is known that AD is a polygenic/complex disorder in which hundreds of genes, distributed across the human genome, might be involved in close cooperation with environmental inducers, cerebrovascular dysfunction, and epigenetic phenomena [1]. The main neuropathological hallmarks of AD include accumulation of amyloid-*β* (A*β*) peptide, neurofibrillary tangle-like structures (NFTs) composed largely of paired helical filaments with phosphorylated tau proteins, neuronal and synaptic loss [2], reduced overall brain volume with specific damage of entorhinal cortex and hippocampus [3], neuroinflammation, gliosis, free radical formation [4], neurotransmitter and neurotrophic factor deficits, metabolic impairment [5], and proteasome and chaperone dysregulation [6]. In the past decade, A*β*-based immunotherapy has been shown to be the most promising therapeutic approach [7–12], and there are over 10,000 patients currently enrolled in active or passive A*β* immunotherapy, showing so far a certain degree of success in decreasing beta amyloid [13–17] and reversing memory deficits [18–20]. However, the first clinical trial in 2001 sponsored by Elan and Wyeth with active immunization, consisting of aggregated synthetic A*β*_42_ peptide delivered in QS21 adjuvant, resulted in 6% patient death due to a meningoencephalitis-like reaction [13, 21], probably induced by an over-extensive T-cell-mediated immune response [22, 23]. Remarkably, patients with abbreviated treatment generated anti-A*β* antibodies, reducing cerebrospinal levels of tau and reported a slower cognitive decline [24, 25]. Prior to clinical trials, numerous experimental therapeutic immunization strategies were developed using transgenic mice models of AD-like pathology, including APPswe/PS1dE9 double transgenic mice, to investigate emergent therapies focused on preventing and/or treating AD neuropathology [26, 27]. Transgenic mice expressing mutated forms of the gene for the human amyloid precursor protein (*hAPP*) prematurely show marked elevation of A*β*-protein levels and deposition in the cerebral cortex and hippocampus [28–30], as seen in the brain of AD patients [31]. Presenilin-1 (*PS1*-) mutant transgenic mice also show an increase in A*β*_1-42_ (A*β*_42_) peptide generation, potentiating amyloid deposition in the brain at about 6 months of age [32]. In the present study we used APPswe/PS1dE9 double-transgenic mice derived from the coexpression of mutated *APP* and *PS1* genes, which have demonstrated a prematurely accelerated accumulation of A*β* deposits in the brain, compared with those expressing singly *APP* or *PS1* mutations alone [33–37]. This feature, together with a variety of other clinically relevant AD-like alterations, points out this model as a valuable tool in the development of new AD therapeutic approaches [38]. In the present study, we present the EB101 vaccine, which is designed to become cost-effective and long-lasting, being targeted toward the reduction of A*β* burden and the slowing of the main AD-like pathological alterations, including the inflammatory reaction, by the induction of an anti-inflammatory T-helper (Th) 2 immune response. All these requirements will be achieved by designing a physiological adjuvant composed of naturally occurring phospholipids, proven safe and efficacious in other type of vaccines, including influenza, with an added biologically active phospholipid, S1P, known to stimulate an anti-inflammatory reaction and act as a neuronal regenerating agent in *in vitro* and *in vivo* studies [39]. 

The purpose of this study was to investigate the effect and safety of an A*β* vaccine (EB101) in APPswe/PS1dE9 transgenic mice elicited by a novel immunogenic adjuvant, designed to reduce A*β* deposition but avoiding the massive activation of T-cell-mediated immune response that potentially caused severe adverse effects. 

## 2. Materials and Methods

### 2.1. Animals

APPswe/PS1dE9 double-transgenic mice (B6C3F1/J), expressing a chimeric mouse/human amyloid precursor protein (Mo/HuAPP695swe) and human presenilin 1 (PS1-dE9) mutants, both directed to neurons of the central nervous system (CNS), were used in this study. These two constructs were coinjected into B6C3HF2 pronuclei and insertion of the transgenes occurred at a single mouse locus. The transgenic mice used were purchased from The Jackson Laboratory. All experimental procedures conformed to the guidelines established by the European Communities Council Directive (86/609/EEC) and by the Spanish Royal Decree 1201/2005 for animal experimentation and were approved by the Ethical Committee of the EuroEspes Biomedical Research Center. 

### 2.2. Experimental Design

Two experimental treatment studies were carried out; one before AD onset, starting at 7 weeks of age (preventive treatment) and the second after AD onset, at 35 weeks of age, when neuropathological AD characteristics were well established (therapeutic treatment). Mice were randomly divided into these two experimental groups, and each group was subdivided into another three, as follows. 

#### 2.2.1. Preventive Treatment

Group A consists of 6 transgenic mice that were immunized with a cocktail of A*β*_42_/S1P/liposome (EB101); group B, 6 transgenic mice immunized with liposomes without A*β*_42_ (EB102); group C, 4 transgenic mice inoculated with vehicle, PBS. Two wild-type mice were also included in each group as a safety immunization control, both in preventive and therapeutic treatments.

#### 2.2.2. Therapeutic Treatment

Group A consists of 6 transgenic mice immunized with EB101; group B, 6 mice immunized with EB102; group C, 4 transgenic mice inoculated with PBS. Mice were immunized with nine injections for seven months and then kept for 2 additional months before sacrifice. Mice were 11 months old at the end of preventive treatment and 18 months old at the end of therapeutic treatment. 

### 2.3. Experimental Procedure

#### 2.3.1. Preparation of A*β*

Two mg of human synthetic A*β*_42_ (TOCRIS bioscience; Tocris Cookson Ltd.) were dissolved in 0.9 mL water, and 0.1 mL of PBS was added. The mixture was vortexed, aliquoted, lyophilized, and stored as a dry powder at −20°C until use.

#### 2.3.2. Preparation of Liposomes

Liposomes were prepared from 1,2-Dioleoyl-sn-Glycero-3-Phosphocholine (DOPC), 1-Palmitoyl-2-oleoyl-sn-glycero-3-phosphatidylglycerol, Sodium Salt (POPG), cholesterol (CH; Northern lipids INC.), plus/minus D-erythro-Sphingosine-1-Phosphate (S1P), (AVANTI at 0.3/0.3/0.39/0.01, molar ratio resp.). One hundred milligrams of each lipid were dissolved in 1 mL of chloroform and stored at −20°C until the preparation of the S1P liposomes, 10 mg was dissolved in 1.5 mL (2 : 1 chloroform/methanol) and stored at −20°C until use. Lipids, in the final proportion indicated above, were thoroughly mixed in the organic solvent, evaporated under nitrogen. The corresponding mixture, containing total lipid 100 mg and 0.6 mg of S1P, was resuspended in autoclaved ultrapure water and thoroughly vortex mixed until a milky solution was formed or the so-called “multilamellar vesicles” (MLVs). Small (SLV) or single unilamellar vesicles (SUVs) were subsequently prepared by sonicating the MLV for 2 minutes at 30-second intervals in an ice bath until the solution was clear and centrifuged at 2500 g/15 minutes to eliminate debris. The SUV preparation was freeze dried and resuspended in PBS-containing A*β* (10 mg) and thoroughly mixed. The freeze-dried mixture was resuspended in the corresponding amount of autoclaved ultrapure water, ready for immunization. 100 *μ*L of EB101 was used for the immunization step. Liposomes containing S1P and A*β* are referred as EB101 and without S1P and Ab are referred as EB102.

#### 2.3.3. Immunization Procedures

Forty-four APPswe/PS1dE9 transgenic mice were inoculated intraperitoneally with 100 *μ*L per injection of EB101 (group A), only liposome complex EB102 (group B) or PBS (group C), during seven months (9 injections) for each treatment period. The immunization protocol was systematically used in previous studies [45, 47], consisting of 3 injections every two weeks, in the first month and then one injection each following month regimen followed in the preventive and therapeutic groups. 

#### 2.3.4. Spleen Cell Preparation

Splenocytes from these mice were harvested from spleen tissue mechanically disrupted with forceps. Lysis of red blood cells was carried out in 100 mM Tris buffer, pH 7.5, containing 140 mM ammonium chloride. Splenocytes were washed twice with PBS pH 7.0 and centrifuged at 1600 rpm for 5 minutes. Cells were counted and resuspended in complete RPMI-1640 medium containing 10% FSC, 50 *μ*M *β*-mercaptoethanol, and 40 *μ*g/mL^−1^ gentamycin at 10^6^ cells/mL. 

#### 2.3.5. Preparation of B-Cell-Enriched Suspensions

B-cell-enriched suspensions were obtained as follows: macrophages from spleen cells were depleted by selective adherence to glass Petri dishes for 2 h at 37°C, and nonadherent cell suspensions were depleted of T cells by magnetic cell sorting using anti-Thy-1.2-coated magnetic beads (Dynal Biotech, France), following the manufacturer's instructions. This procedure yielded an enriched B-cell population of >90% CD19^+^ cells with <1% CD3^+^ cells and <5% CD11c^+^ cells, as determined by flow cytometry analysis, with >95% viable cells, as determined by trypan blue exclusion. 

#### 2.3.6. B- and T-Cell Phenotypic Analysis Was Carried Out by Flow Cytometry

Briefly, 1 × 10^6^ red-cell-depleted splenocytes were incubated with anti-mouse CD32/CD16 antibody (control antibody) to block nonspecific Ig trapping through Fc receptors. The following PE-conjugated anti-mouse mAbs were used for analysis: anti-CD4, anti-CD19, anti-IgM, and anti-CD138 (BD PharMingen, San Diego, CA, USA). Data from stained samples were acquired using FACS flow cytometry. A total of 15,000 events were analyzed using Lysis II software.

#### 2.3.7. Lymphoproliferation Assay

Proliferation assays were set up in triplicate in 96-well flat bottom plates (Nunc, Naperville, IL, USA). Each culture consisted of 1 × 10^5^ purified B and T cells in a final volume of 200 *μ*L and incubated with LPS (20 *μ*g/mL^−1^), IL-4 (100 U/mL^−1^), anti-CD40 (5 *μ*g/mL^−1^), and A*β* (1 *μ*g/mL). After 48 h incubation at 37°C in 5% CO_2_, the proliferative response was monitored by MTT assay, based on the cleavage of the yellow tetrazolium salt MTT to purple formazan crystal by metabolic active cells. The formazan was then solubilized and the concentration determined by optical density at 570 nm. Cells were seeded into 96-well tissue culture plates at a density of approximately 5000 cells per well. After stimulation, 10 *μ*L of MTT solution was added to each well. Plates were mixed by briefly shaking on an orbital shaker and incubated at 37°C for 4 hours. Medium was removed and 200 *μ*L DMSO was added into each well to dissolve the formazan. Absorbances were measured in an ELISA plate reader at a wavelength of 570 nm. The results obtained from triplicate assays were expressed as stimulation indexes (ratio between mean OD from stimulated cultures and mean OD from unstimulated ones). Values are representative of at least three independent experiments.

#### 2.3.8. Measurement of Antibody Titers

96-well microtiter plates (Costar EIA plates) were coated with 1 *μ*g/mL of A*β*_1-42_ in well-coating buffer (0.1 M sodium phosphate, pH 8.5, 0.1% sodium azide) and incubated overnight at room temperature (RT). The wells were aspirated and sera added to the wells at a starting dilution of 1/100 in specimen diluent (0.014 M sodium phosphate, pH 7.4, 0.15 M NaCl, 0.6% bovine serum albumin, 0.05% thimerosal). Six serial dilutions (1/400, 1/1.600, 1/6.400, 1/25.600), of the samples were made directly in the plates in twofold steps to reach a final dilution of 1/102.400. The samples were incubated in the coated wells for one hour at RT. The plates were then washed five times with PBS containing 0.05% Tween 20, and a second antibody, a goat anti-mouse Ig conjugated to horseradish peroxidase (obtained from Boehringer Mannheim), was added to the wells (100 *μ*L/well at a dilution of 1/4.000 in specimen diluent) and incubated for one hour at RT. Plates were again washed five times in PBS/Tween 20 and developed with chromogen, 100 *μ*L TMB (3,3,5,5′-tetramethyl benzidine obtained from Pierce Chemicals), which was added to each well and incubated for 15 minutes at RT. The reaction was stopped by the addition of 50 *μ*L 0.5 M H_2_SO_4_. The color intensity was then read on an ELISA plate reader (BioRad 680 ELISA Reader). Titers were defined as the reciprocal of the dilution of serum giving one half the maximum OD. Maximal OD was generally taken from an initial 1/100 dilution. 

### 2.4. Immunohistochemistry

Animals under anesthesia were perfused transcardially with NaCl solution followed by 4% paraformaldehyde. Their brains were then excised and immersed in paraformaldehyde for 48 hrs, followed by immersion in phosphate buffer 0.1 M for 12 hrs and cryoprotected with 30% sucrose in PB, embedded in OCT compound (Tissue Tek, Torrance, CA, USA) and frozen with liquid nitrogen-cooled isopentane. Parallel series of transverse sections (18/20 *μ*m thick) were cut on cryostat and mounted on Superfrost Plus (Menzel Glasser, Madison, WI, USA) slides. For immunohistochemical analysis, parallel sections were pretreated with H_2_O_2_ in PBS at 37°C for 15 minutes to eliminate endogenous peroxidase, rinsed twice in 0.05 M Trizma buffered saline (TBS) containing 0.1% Tween-20 at pH 7.4 (TBS-T) for 10 minutes each, pretreated with blocking avidin/biotin kit and then incubated overnight with the primary antibodies (Table 1). The procedure provided by the mouse-on-mouse peroxidase immunodetection system (M.O.M. Kit; Vector) was used to eliminate any nonspecific binding of anti-mouse secondary antibodies with the endogenous mouse immunoglobulins in the tissue, according to the manufacturer's instructions. The sections were successively rinsed in TBS-T, incubated in goat IgG anti-rabbit (Dako) or goat IgG anti-mouse (Dako) for 1 hr, rinsed in TBS-T, and then incubated for 30 minutes in ABC kit system (Vectastain, Vector). Peroxidase reaction was performed with 3,3-diaminobenzidine as chromogen and hydrogen peroxide as oxidant. In several adjacent sections, negative controls performed by omitting the primary, secondary, or tertiary antibodies showed no immunostaining. Sections were then dehydrated in graded ethanol and covered with a rapid embedding agent (Eukitt, Fluka). 

### 2.5. Neuropathology Marker Quantification

**Figure 1:**
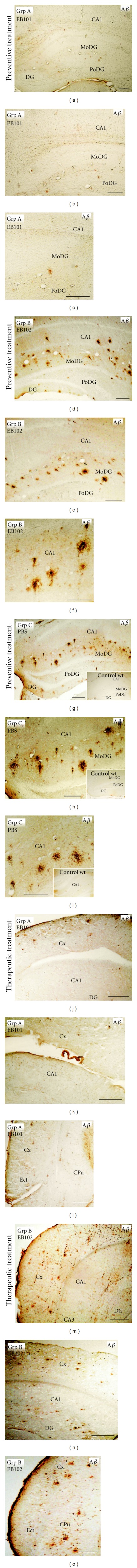
Effect of EB101 vaccine on beta amyloid (A*β*) deposits in the brains of B6C3F1/J mice. Comparative photomicrographs of A*β* immunoreactivity were taken in the hippocampus ((a)–(k), (m), (n)) and cortical ((l), (o)) brain regions of transgenic mice treated with the vaccine (Grp A) before A*β* plaques develop preventive treatment ((a)–(i)) and after the A*β* plaques developed therapeutic treatment ((j)–(o)). *Preventive Treatment*: Transverse brain sections of 11-month-old mice show almost complete absence of A*β* deposits in the dentate gyrus ((a)–(c)) and hippocampal subregion CA1 following EB101 vaccine immunization (group A), contrasting sharply with the numerous A*β* immunoreactive plaques in the corresponding mouse brain sections treated with EB102 (group B in (d)–(f)) or PBS (group C in (g)–(i)). Immunoreactive A*β* in the brain sections of wild-type mice is absent (inserts in (g)–(i)). *Therapeutic Treatment*: Transverse brain sections of 18-month-old mice are shown in (j)–(o). Treatment with EB101 almost completely abolished A*β* load in the retrosplenial cortex/hippocampal subregion CA1 ((j)–(k)) and ectorhinal cortex (l) compared to the same areas of mice treated with EB102 ((m)–(o)), as determined by number of plaques and staining intensity area. Scale bar: 100 *μ*m.

**Figure 2:**
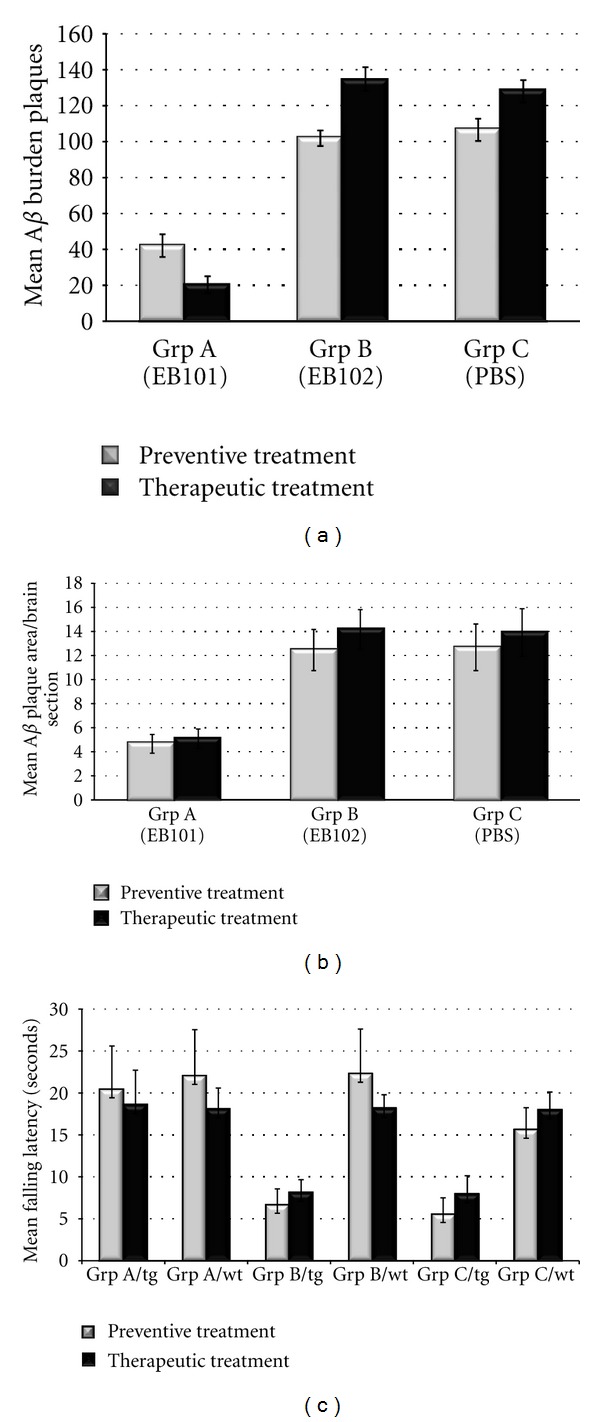
Analysis of A*β* burden in the brain and motor coordination of B6C3F1/J mice. (a) Mean of A*β* burden in the hippocampal and cortical regions of APPswe/PS1dE9 mice in the three treatment groups (EB101, EB102, and PBS). The mean of the A*β* burden is reduced in group A (EB101) when compared with group B (EB102) and group C (PBS) during preventive treatment and markedly reduced in the therapeutic treatment period. Data are presented as mean ± standard error of the mean (SEM). (b) Quantitative analysis of A*β* burden area in the hippocampal and cortical equivalent regions of APPswe/PS1dE9 mice treated with EB101, EB102, and PBS, represented by the number of pixels inside the stained area of each A*β* plaque. Four randomly selected mouse brain sections of preventive and therapeutic treatment groups were assessed using Pixcavator (version 4) analysis software. This figure shows that A*β* plaques of EB101-treated mice are significantly smaller in size than those of the other two treatment groups (EB102 or PBS). Data are presented as mean ± SEM. (c) Rota rod test results of motor abilities after the preventive and therapeutic treatment show no negative effect of EB101 compared to EB102- and PBS-treated mice. Wild-type mice showed no significant differences among treatment groups. Values represent the means of all six trials of a group/day (*P* ≤ 0.01).

A*β* plaque quantification was determined in 7 randomly selected microscopic transverse sections per treated animal (Figures 1 and 2), as defined by the stereotaxic Bregma coordinates (−0.94; −1.34; −1.70; −2.30; −2.70; −3.64; −4.60 mm). A total of 7 selected sections per animal were evaluated on a PC using the HIH Image J program by defining region of interest and setting a threshold to discriminate nonspecific staining. The same procedure was undertaken for the neurofibrillary tangle-like structure sections and activated astroglia, B and T cells. All data analysis and measurements were blindly performed by investigators unaware of the treatment protocol, and in cases of significant measurement discrepancies between two investigators, the evaluation was repeated by a third. Quantitative analysis of amyloid burden area was also performed in the hippocampal regions (oriens, pyramidal layer, stratum radiatum, and dentate gyrus) and parietal/temporal cortical regions in the three experimental groups (Figures 1 and 2). Area/pixel analysis software (Pixcavator 4) was used to quantify the number of pixels inside the outer boundary of each A*β* plaque for one brain section. Thus, Pixcavator imaging was used to analyze the area occupied by *β*-amyloid (A*β* load) relative to the background and expressed in percentage units. The area of A*β* plaques of the brain of three treatment groups is represented in graphics (Figure 2). 

### 2.6. Imaging

Images were visualized using a microscope (Olympus BX50) and digitized using a digital camera (DP-10, Olympus). The photographs were adjusted for brightness and contrast with Corel Photo-Paint (Corel, Ottawa, Canada) and plates were composed with Corel Draw.

#### 2.6.1. Confocal Imaging Analysis

Each individual immunofluorescent section was photographed with a Spectral Confocal Laser Scanning Microscope (Leica TCS-SP2). Plaques were imaged at the level of their largest cross-section, and their size was determined by using the Leica processing software. This methodological approach was used to compare NFTs-A*β*, NFTs-GFAP, and NFTs-Bcell immunostainings. Two brain regions (hippocampus and entorhinal cortex) per animal were analyzed. Imaging of the NFTs immunostaining was revealed with a fluorescein isothiocyanate filter (excitation at 488 nm), and A*β*/GFAP/B-cell staining was imaged with a Texas Red filter (excitation 568 nm).

### 2.7. Evaluation of Motor Coordination and Balance

The motor strength, ability, balance, and coordination skills of all the experimental mice in the two treatment periods were evaluated in a rota-rod operated at 10 rpm (Columbus Instruments, Columbus, OH, USA) beginning at 7 weeks of age (preventive group) and 35 weeks of age (therapeutic group), respectively. The animals were adapted to the apparatus by receiving training sessions in two trials, sufficient to reach a baseline level of performance [48]. This procedure was designed to assess motor behavior. If the mouse remained on the rod for 1 minute, the test was completed and scored as 1 minute. Each animal was tested during six weeks for three sessions, with each session separated by 15 minutes, the time each mouse remained on the rod was registered automatically and stopped when the animals fell or inverted (by climbing) from the top of the rotating barrel. Two investigators performed the experiment, one evaluated rota-rod performance, unaware of the mice treatments, and the second treated the mice. 

### 2.8. Statistical Analyses

All statistical parameters were performed by SPSS (version 11.0; SPSS Inc, Chicago); a *P* value < 0.05  indicated statistical significance. Average range of A*β* plaque density, burden area, and antibody titers developed by mice during treatments were analyzed using a two-factor repeated-measures analysis of variance (ANOVA) followed by a *post hoc* analysis when relevant. All data were expressed as the mean ± SEM.

### 2.9. Limitations of the Study

Preclinical research in AD is linked to the use of a wide range of transgenic mouse models that may recapitulate some of the pathological features of clinical human AD. Despite the fact that to date, none of the models developed fully the entire disease background [38]; the authors believe that APPswe/PS1dE9 transgenic mice present the right pathological features to answer the specific questions addressed by the study. Another issue is the possible disadvantages of an active A*β* immunotherapy. Although its potential to be more cost effective and long-lasting and to yield efficient results in reducing AD-like manifestations is known, some aspects, such as an undesirable immune response induced by the adjuvant, long immune response adjustment, age-dependency, inflammation, and microhemorrhages, have to be considered. Since the aim of this study was to assess the effect of EB101 vaccine in the neuropathological hallmarks of APP/PS1 mice, memory function tests were not performed although they will be included in future studies. However, motor coordination tests have been performed in order to validate the locomotion integrity during the entire immunotherapeutic process, as reported in similar previous studies. The authors, being aware of these issues, designed the present study aiming to solve and overcome these disadvantages.

## 3. Results

### 3.1. Prophylactic Effects of EB101 on Alzheimer-Like Pathology in APPswe/PS1dE9 tg Mice

Amyloid plaques were present in the brain of all mice except wild-type controls (Figures 1(a)–1(o)), although the density and the burden area of the A*β* deposits were substantially higher in mice of group B (Figures 1(d)–1(f)) and C (Figures 1(g)–1(i)). Image analysis of brain sections indicated a considerable reduction of the median burden value of A*β* deposits in the hippocampus and cortical layers of EB101-treated transgenic mice (group A; 42.5 A*β* plaques/brain) when compared with mice treated with EB102 (group B; 102.2 A*β* plaques/brain) and PBS (group C; 106.9 A*β* plaques/brain) (Figure 2(a)). In each brain section of EB101-treated transgenic mice, we observed fewer A*β* deposits, mainly located at the external layers of the dentate gyrus (Figures 1(a)–1(c)), representing plaques of an early-stage-like morphology characterized by tiny sparse plaques with diffuse core staining (type 2a). The histological analysis of mouse brain sections revealed four different types of A*β* plaques: type 1, 2a, b, and c, based on the morphological characterization described by Bussière et al. [44], as observed in Figures 1(a)–1(i). These *β*-amyloid plaques were mainly present in the hippocampus of transgenic mouse brains of groups B and C (Figures 1(d)–1(i)), located mainly in the dentate gyrus (granular layer), followed by neocortical regions such as retrosplenial areas, ectorhinal cortex, and piriform cortex layers. The area occupied by A*β* deposits in both the hippocampus and cerebral cortex of transgenic mouse brain sections of group A (EB101; Figures 1(a)–1(c)) after preventive treatment was markedly reduced (4.83%, *P* < 0.05; Figure 2(b)) and differed significantly from the elevated A*β* deposit area observed in the correspondent brain sections of group B (12.6%, *P* < 0.05; Figure 2(b)) and group C (12.81%, *P* < 0.05; Figure 2(b)). All wild-type mice of each group showed no A*β* deposits in any brain region (see squared area in Figures 1(g)–1(i)). 

The balance, coordination, and motor planning in this mouse study were assessed at the end of the 7-month treatment using a rota-rod test. Results of motor abilities showed an improvement in motor coordination in the EB101-treated mice compared to the two other controls, which showed a moderate-to-severe impaired motor coordination (Figure 2(c)). 

**Figure 3:**
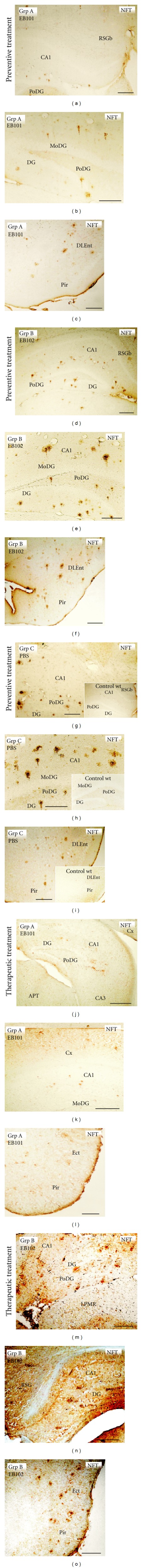
Effect of EB101 vaccine on neurofibrillary tangle-like structures in the brains of B6C3F1/J mice. Comparative photomicrographs of neurofibrillary tangle-like structure immunoreactivity taken in the hippocampus ((a), (b), (d), (e), (g), (h), (j), (k), (m), (n)) and cortical ((c), (f), (i), (l), (o)) brain regions in the preventive treatment group, ((a)–(i)) and therapeutic treatment group, ((j)–(o)). *Preventive Treatment*: Transverse brain sections of 11-month-old mice show almost complete prevention of neurofibrillary tangle-like structure (NFTs) formation after EB101 vaccine immunization in the hippocampal subregion CA1 (a), dentate gyrus (b), and entorhinal cortex (c) contrasting with the same regions of EB102-immunized mice ((d)–(f)) and PBS ((g)–(i)). Immunoreactive NFTs are absent in the brain sections of wild-type mice (inserts in (g)–(i)). *Therapeutic Treatment*: Transverse brain sections of 18-month-old mice show almost complete reduction of NFTs after EB101 vaccine immunization in the dentate gyrus (j), hippocampal subregion CA1 (k), and entorhinal cortex (l), compared with the same regions of EB102-immunized mice ((m)–(o)). Scale bar: 100 *μ*m.

The effect of the EB101 vaccine on the development of neurofibrillary tangle-like structures (NFTs) was also studied using specific antibody against NFTs (Table 1). Stained NFTs were observed in all transgenic mice except wild-type controls (Figures 3(a)–3(i)). Similar to that observed with A*β* deposits, the density of these stained tangles was substantially higher in mouse brains of group B (Figures 3(d)–3(f)) and C (Figures 3(g)–3(i)) compared to the EB101-treated group A. These immunopositive structures were mainly located in the hippocampal brain regions of the mice treated with EB102 and PBS and were composed of aggregated hyperphosphorylated tau protein located along the neuronal helical filaments with a plaque-like immunoreactive core and an apical variable dendrite extension (often having a flame-shape appearance, Figure 3). The brain regions of mice treated with EB101 (group A) were mostly devoid of NFTs (Figures 3(a)–3(c)), with scattered immunoreactive tangles in the dentate gyrus (Figure 3(b)) and entorhinal cortex layers (Figure 3(c)). The same regions of group B (Figures 3(d)–3(f)) were notably similar to those observed in mice of group C (Figures 3(g)–3(i)) and showed an extensive density of NFTs occupying all hippocampal regions, retrosplenial areas, ectorhinal, and piriform cortex. The stained intensity of the NFTs observed in groups B and C, including density and burden area, contrasts markedly with the scarcity and almost absence of these neuropathological features in mice treated with EB101. 

### 3.2. Therapeutic Effects of EB101 on Alzheimer-Like Pathology in APPswe/PS1dE9 tg Mice

The therapeutic effect of EB101 vaccine in APPswe/PS1dE9 tg mice was initiated in 35-week-old mice after the appearance of the Alzheimer-like neuropathology in defined brain regions. To determine whether EB101 vaccine reverses the massive development of *β*-amyloid plaque, NFTs and reactive glia, brain sections from wild-type and transgenic mice of all experimental groups were immunostained with their specific antibodies (Table 1). The results obtained after the therapeutic treatment showed that A*β* deposits were almost absent in brain sections of the EB101-treated mice of group A (20.3 A*β* plaques/brain) (Figures 1(j)–1(l) and 2(a)), markedly different from the A*β* burden levels observed in the mice treated with EB102 (134.3 A*β* plaques/brain; Figures 1(m)–1(o) and 2(a)) and PBS (128.6 A*β* plaques/brain). The few A*β* plaques observed in group A mice presented a tiny central core surrounded by scarce fibrillar material (type 2a), with lower A*β* deposit area (5.27%, *P* < 0.05; Figure 2(b)), mainly located at the external cortical layers (Figures 1(j)–1(l)) and almost totally absent in the hippocampal regions. In the brain section of the same transverse regions, group B mice presented an extensive A*β* plaque area (14.32%, *P* < 0.05; Figures 1(m)–1(o) and 2(b)), showing a scattered distribution throughout the hippocampal and cortical layers, similar to that observed in group C (14.07%, *P* < 0.05; Figure 2(b)). 

The rota-rod latency test, addressing motor coordination during the therapeutic treatment, showed no negative performance by the EB101-treated-group (Figure 2(c)), but mice treated with EB102 and PBS (Figure 2(c)) showed moderately-to-severely impaired motor abilities and coordination. 

Histological analysis of mice in group A showed a less dense distribution pattern of NFTs (Figures 3(j)–3(l)) than that observed in the preventive treatment. However, mice from group B showed a similarly elevated density of NFTs (Figures 3(m)–3(o)) to those seen in mice in the preventive treatment. 

### 3.3. Double Immunofluorescence Detection of Neuropathological Markers in APPswe/PS1dE9 tg Mice

**Figure 4:**
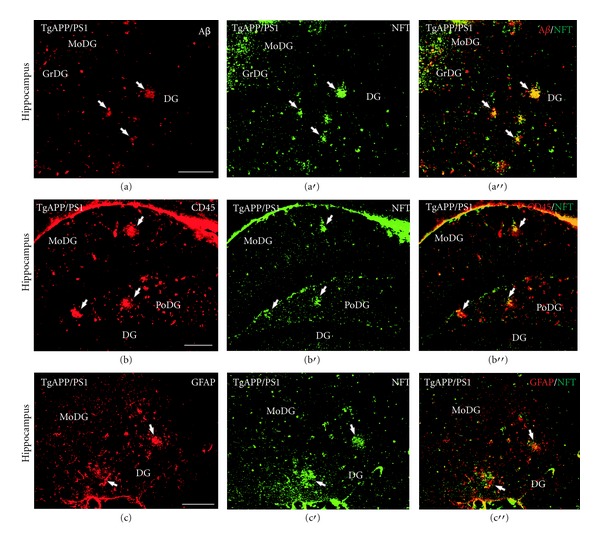
Association of neurofibrillary tangle-like structures with activated microglia, A*β* deposits, and B cells in B6C3F1/J mouse brains. Photomicrographs of transverse sections of hippocampal brain regions of 11-month-old double-transgenic APP/PS1 mice show codistribution of immunofluorescent reactivity to NFTs, A*β*, and GFAP. ((a)–(a′′)): Photomicrographs of transverse brain sections at the hippocampus show immunofluorescence to A*β* (red), NFTs (green), and merged channels (yellow). Note some double-labeled sparse plaques (colocalization showed by white arrows) immunofluorescence to both A*β*/NFTs antibodies (yellow) at the molecular layer of the dentate gyrus (a′′). ((b)–(b′′)): Photomicrographs of transverse brain sections at hippocampus show immunofluorescence to CD45-B cells (red), NFTs (green), and merged channels (yellow). Note some codistribution of B cells and NFTs (yellow) in this region (white arrow in (b′′)) where NFTs (white arrow in (b′)) are surrounded by some B cells (white arrows in (b)). ((c)–(c′′)): Photomicrographs of transverse brain sections at hippocampus show immunofluorescence to GFAP (red), NFTs (green), and merged channels (yellow). Note glial cells (white arrows in (c)) surrounding the NFTs (white arrows in (c′)) at the dentate gyrus, although no colocalization (yellow) was observed (c′′). For abbreviations, see list. Scale bar: 100 *μ*m.

Simultaneous double immunofluorescence was performed to detect colocalization of A*β* plaques and neurofibrillary tangle-like structures in mouse brains (Figures 4(a)–4(a′′)). Some sparse plaques in the hippocampus and entorhinal regions of mice were observed, colocalized in the molecular layer of the dentate gyrus (Figures 4(a)–4(a′′)) and at the entorhinal cortex. Immunofluorescence to CD45RA (B cells) and NFTs was also analyzed by confocal microscopy, showing B cells surrounding the NFTs where some codistribution was observed in the polymorph and molecular layers of the dentate gyrus (Figures 4(b)–4(b′′)). Double immunofluorescence to GFAP and NFTs showed that glial cells were surrounding NFTs in the dentate gyrus (Figures 4(c)–4(c′′)) and entorhinal cortex, although no colocalization was observed.

### 3.4. Effect of EB101 on the Immune Response

**Figure 5:**
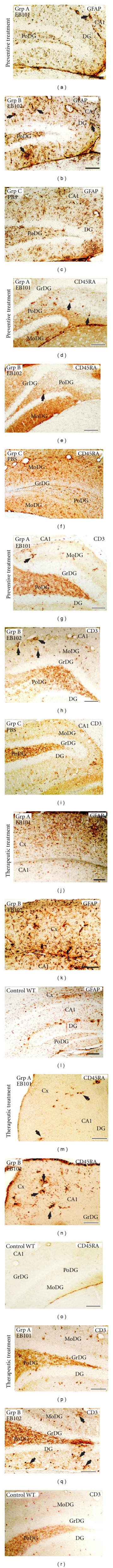
Effect of EB101 vaccine on astrocytosis and activation of B and T cells in the brains of B6C3F1/J mice. Comparative photomicrographs of GFAP, CD45RA, and CD3 immunoreactivity in the hippocampus ((a)–(i), (l), (o), (r)) and cortical ((j), (k), (m), (n), (p), (q)) brain regions in the preventive treatment group ((a)–(i)) and therapeutic treatment group ((j)–(r)).

To assess the effect of immunization on glial reactivity (astrocytosis) and active lymphocytes, the distribution of glial fibrillary acidic protein (GFAP), B-cell (CD45RA) and T-cell (CD3) surface markers was analyzed in the transverse section of the mouse brains (Figures 5(a)–5(r)). After preventive treatment, activated or reactive glia, astrocytosis-like morphology with aggregated astrocytes replacing dead neurons was observed surrounding A*β* plaques and NFTs in the cortical and hippocampal brain regions of transgenic mice of groups B (Figure 5(b)) and C (Figure 5(c)). The same regions of transgenic mouse brains of group A were almost devoid of reactive glia (Figure 5(a)). No astrocytosis was observed in wild-type mice (Figure 5(l)). Brain sections of EB101-treated transgenic mice showed some immunoreactive B cells in the hippocampal regions (Figure 5(d)), with much lower density in groups B (EB102; Figure 5(e)) and C (PBS; Figure 5(f)). No significant B-cell or T-cell aggregates were observed in any of the wild-type mouse brain sections (Figure 5(o)). In the preventive treatment, immunoreactive T cells were found in all transgenic mouse brains, forming conspicuous aggregates in the hippocampal (Figures 5(g)–5(i)) and cortical regions, although these lymphocyte clusters were smaller in the EB101-treated transgenic mice (Figure 5(g)). 

After therapeutic treatment, glial distribution in transgenic mouse brains treated with EB101 (Figure 5(j)) had a similar pattern to wild-type mice, with no reactive glia throughout the brain except conspicuously in specific cortical areas. The overall GFAP immunoreactive cell distribution pattern showed no reactive astrocytosis in group A treated with EB101, contrasting with groups B (Figure 5(k)) and C, where prominent GFAP-associated immunoreactivity was present in the hippocampal and cortical regions. B-cell density in the EB101-treated group (Figure 5(m)) after therapeutic treatment was similar to the preventive regime. However, groups B (Figure 5(n)) and C showed a pronounced increase in CD45RA surface markers in the cortical brain areas with dense fibrillar A*β*-containing plaques. Groups B (Figure 5(p)) and C showed numerous clustered T-cells in hippocampus and neocortex, consisting of CD3-immunoreactive cells in areas rich in amyloid plaques (Figure 5(q); see also double staining results in Figure 4). T-cell microglia was practically undetected in EB101-treated group (Figure 5(p)), similar to that observed in wild-type controls (Figure 5(r)). 

From the present data related to experiments shown in Figure 5, the following observations are derived from the two treatment groups performed in our studies.

*Preventive Treatment: *Transverse brain sections of 11 mo mice treated with EB101 show almost a total absence of astrocytosis in the dentate gyrus (a), contrasting with densely dystrophic reactive astrocytes in the corresponding mouse brain sections of groups B (EB102-treated mice; (b)) and C (PBS-treated; (c)). Detailed section of the hippocampus shows an abundant density of B-ir cells in EB101 immunized mice (d) compared with EB102 and PBS treated mice ((e)-(f)), and practically absent in the wild-type brain sections. Transverse hippocampal sections showing a significant reduction in T-cell aggregation (g) compared with mice immunized with EB102 (h), where numerous microglial clusters with B-ir cells, are observed mainly in the outer layers of the dentate gyrus. Scarce T-ir cells were observed in the brain sections of wild-type mice (i).

*Therapeutic Treatment: *Transverse brain sections of 18 mo mice show an almost complete reduction of astrocytosis in the retrosplenial cortex/hippocampal subregion CA1 (j) after EB101 vaccine immunization. Note the contrast between EB101- and EB102-immunized mice (k) and absent in the brain sections of wild-type mice (l). Moderate density of B-ir cells is observed in transverse cortical/CA1 regions of transgenic mice treated with EB101 (m), contrasting markedly with the massive immunoreactive clusters of B cells observed in EB102-immunized mice (n), indicative of a neuropathological inflammation process. B-ir cells are absent in the brain sections of wild-type mice (o). Transverse hippocampal sections of EB101-treated mice show scarce density of T-ir cells in the dentate gyrus (p), contrasting with a significant increase in the same brain area of EB102-immunized mice (q). T-ir cells in the brain sections of wild-type mice are scarce (r). For abbreviations, see list. Scale bar: 100 *μ*m.

### 3.5. A*β* Antibody Titers in APPswe/PS1dE9 Transgenic Mice

**Figure 6:**
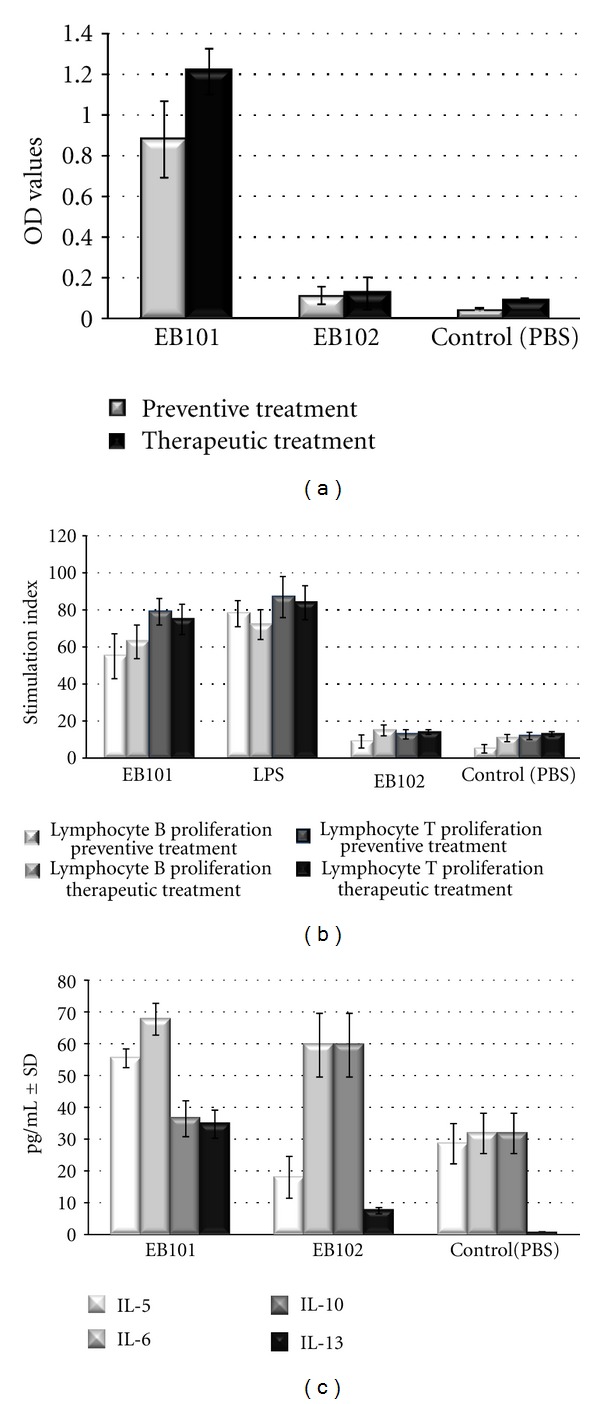
Quantification of IgG antibodies against beta-amyloid and Th1/Th2 cytokine detection after immunization. (a) Presence of anti-*β*-amyloid in the sera of EB101-vaccinated and control transgenic mice groups after preventive and therapeutic treatment. EB101 led to a quite high increase of IgG antibody production as shown. ELISA was used for sera antibody detection in mice in each group. Each bar represents the background OD mean value ± SEM in each group. (b) Proliferative response of spleen cells from EB101-treated mice and controls after preventive and therapeutic treatment. Results showed that both EB101 and LPS induced B and T lymphocyte proliferative responses. Note that EB101 treatment resulted in an enhanced proliferation response when compared with EB102 and PBS control mouse groups (*P* ≤ 0.01). The results obtained from triplicate assays were expressed as stimulation indices (ratio between mean OD ± SEM from stimulated and unstimulated cultures). (c) Detection of Th1 and Th2 cytokines in transgenic mice after therapeutic treatment. Analysis of variance (ANOVA) for IL-5, -6, -10, and -13 indicated significant differences between vaccinated and control groups. Results are shown as mean pg/mL ± SEM.

Preventive treatment with EB101 resulted in a marked increase in IgG A*β*_42_ antibody production in 5 out of 6 animals. Four of 5 immunized mice developed and maintained serum antibody titers between 1 : 1400 and 1 : 2000 and 1 : 6000 in the fifth mouse. No antibodies were detected in control groups B and C. After therapeutic treatment, all mice treated with EB101 produced IgG antibodies (Figure 6(a)). Titers were similar to those detected after the preventive treatment; one out of 6 mice showed titers higher than 1/35,000. In both preventive and therapeutic groups, EB101 induced a strong lymphocyte proliferative response of B- and T cells (Figure 6(b)), while practically no response was observed in EB102- and PBS-treated groups. Data obtained from the lymphoproliferation assay indicated that EB101 resulted in an enhanced proliferative response, compared with EB102 and PBS control groups (*P* < 0.01; Figure 6(b)).

Detection of Th1 and Th2 cytokine types in all treated mice was carried out by analyzing the cytokine profile in mice sera (Figure 6(c)). Results from the multiple analyte detection indicate significant differences between vaccinated and control groups (EB102 and PBS) in sera cytokines IL-5 and IL13, whereas IL-6 and IL-10 showed significant differences between vaccinated and the control group, suggesting that the antibody-mediated immunity observed in the present study induces a T-cell type Th2 reaction.

## 4. Discussion

We have developed a novel anti-A*ß *liposome-formulated vaccine that is cost effective, with long-lasting effects and that not only induces a strong anti-A*ß *antibody reaction, but also decreases neurofibrillary tangle-like structure formation and the inflammatory immune response previously seen with other vaccines. Driven by the great effective potential of liposome-based vaccines in preventing or treating AD [54] and the effectiveness of the first active vaccine in reducing A*ß *levels [21], we have undertaken a new approach to circumvent the AN1792 failure by judiciously selecting an adjuvant that addresses all the above targets. The liposomal adjuvant used consisted of naturally occurring phospholipids: phosphatidyl choline, phosphatidyl glycerol, and cholesterol, proven safe and efficacious in other types of vaccines. To this phospholipid mixture, a biologically active sphingolipid, sphingosine-1-phosphate (S1P) was added. A*ß* was incorporated in the phospholipid/S1P-liposomes using the hydration-rehydration method commonly used for liposomal/protein formulation. S1P, a phosphorylated product of sphingosine, has been implicated as an important lipid mediator acting both inside and outside the cells [55, 56]. Extracellularly, S1P binds to members of the GTP-binding protein (G-protein-) coupled S1P receptor family (S1P15), triggering diverse cellular effects including angiogenesis, cardiac development, immunity, cell motility, and neurite42 extension [25, 57], by acting in an autocrine and paracrine manner [53–55]. Intracellularly, S1P has been shown to function by mediating mobilization of cellular calcium, cell growth, and suppression of apoptosis [56–58], while exogenously-added S1P itself causes glutamate secretion from presynaptic sites and potentiates glutamate-induced transmitter secretion in primary hippocampal neurons [64], which may facilitate the formation of a positive activation cycle in excitatory neurons such as glutaminergic neurons. In fact, S1P appears to have a therapeutic potential as a regenerative agent in the nervous system since it has recently been reported that intracellular S1P enhances nerve growth factor-induced excitability in rat sensory neurons [65]and controls migration of neuronal stem cells toward a site of spinal cord injury as well [66]. Moreover, S1P1activation is also known to induce cytoskeletal rearrangements through small G-protein Rac activation [67], which seems to facilitate synaptic vesicle fusion to plasma membranes inducing transmitter secretion. Therefore, it would not be unexpected that such a potent biologically active sphingolipid may play a very important role in neuronal regeneration, cell growth, suppression of apoptosis, and glutamate secretion from presynaptic sites in AD patients. Taking advantage of these properties, we incorporated S1P with the phospholipid mixture to form a liposomal matrix used as adjuvant to deliver the active antigen, A*ß* [68]. This adjuvant added regenerative and anti-inflammatory properties to the A*ß*42 vaccine, having a considerable advantage over the previously developed saponin-based A*ß* vaccines, key elements to increase neuronal activity and prevent inflammation in the brain of APPswe/PS1dE9 transgenic mice (data not published), improving the previously reported A*ß*-based immunotherapy studies [7–17]. This new vaccine overcame the inflammatory response encountered with AN1792, which potentially was due to the use of a saponin adjuvant (QS-21) and the detergent polysorbate 80 used to deliver A*ß*, which are believed to have induced a proinflammatory Th1 response [33]. We have shown that EB101, a liposomal-based vaccine formulation, fulfills a long-standing need for a safe and42 effective therapeutic vaccine for preventing or ameliorating AD-like neuropathology, improving the beneficial effects reported in previous similar studies [7, 10, 19]. The results observed in the preventive and therapeutic treatments indicate that EB101 vaccine is effective not only in preventing AD-like neuropathology but also in reducing it. Previous APPswe/PS1dE9 transgenic mouse studies reported that in the first 6 months, brain A*ß *plaques were tiny but compact, increasing with age [31, 69]. Our results are also consistent with these observations. APPswe/PS1dE9 transgenic mice showed tiny, compact nonfibrillary amyloid plaque accumulation in the initial deposition period, mainly at the cortex and hippocampus, whereas at later stages diffuse fibrillar plaques were increasingly represented also at nearby brain regions. After the preventive immunization with EB101, we detected a significantly low density of A*ß *plaques in APPswe/PS1dE9 mouse brains, as well as reduced burden areas when compared with APPswe/PS1dE9 mice treated with EB102 or PBS. The fact that image analysis confirmed this efficient reduction of early A*ß *deposits in the EB101 mouse brain suggests that EB101 vaccine acts as a preventive treatment in the onset of the amyloid plaques of Alzheimer's disease in mice. A similar preventive immunization approach has been already reported in PDAPP mice by Schenk et al. [7], although differences in the immunization efficiency and vaccine conformation have been improved in the present study. During the early period, immunization with EB101 also significantly prevented the massive development of other AD pathological hallmarks such as neurofibrillary tangle-like structures, astrocyte activation, and impairment in motor coordination. One of the keys in the success of a preventive immunization is the hindrance of massive glial activation in response to early development of A*ß *deposits [12, 13, 35, 64–66]. The present data from our preventive immunization protocol indicate that when compared with EB102 and PBS treated mouse groups, EB101 also prevents the development of astrocyte activation. Since microglia and astrocyte activation has been reported to degrade A*ß* [69, 72, 73], the near absence of A*ß*-related astrocytosis in EB101-treated mice confirmed the preventive effect on the development of AD-like pathology in this mouse model. Results from the double-staining brain tissues of APPswe/PS1dE9 mice also demonstrated this pathological interaction, since a large number of GFAP-positive astrocytes surrounding neurofibrillary tangle-like structures, closely related to the amyloid plaques and neuroinflammation markers, consistent with previous reports [49, 74, 75]. Taken together, the EB101 vaccine significantly prevents the accumulation, in the initial deposition period, of nonfibrillary amyloidplaques and an astrogliosis reaction background at the cortex and hippocampus of APPswe/PS1dE9 mice. The majority of the related literature has focused on the therapeutic aspects of A*ß *immunization [76]. While A*ß *immunotherapy presently represents the most promising approach for the treatment of AD, one adverse effect is the occurrence of microhemorrhages as described in APP transgenic mice following passive [16, 77] or active A*ß *immunization [16], including that seen with AN1792 during clinical trials [21, 78]. No microhemorrhages however, were observed in APPswe/PS1dE9 mice treated with EB101. The most striking beneficial effect of A*ß *immunotherapy observed in previous studies is the reduction of amyloid burden in AD mouse models [7–19, 26, 65]. This effect was also observed in the present study, where the results after the therapeutic treatment clearly showed that EB101 can reduce and reverse A*ß *plaques and the associated pathological hallmarks. Immunohistochemical and ELISA analysis demonstrated a strong effect of EB101 treatment on A*ß *burden clearance, elevated anti-A*ß *titer levels, near absence of astrocytosis and a normal motor coordination tasks when compared with EB102- and PBS-treated mice. In order to address the complete effect of EB101, we also performed motor-coordination tests instead of memory tasks, since previous reports [37] assumed that this mouse model exhibits detectable behavior changes concurrent with plaque deposition, and that A*ß *pathology across different ages did not correlate with synaptic and cognitive deficits, suggesting that A*ß *levels are not a marker of memory decline. Results of motor abilities showed an improvement in motor coordination in the EB101-treated mice compared to the two other control groups, which showed a moderate-to severe impaired motor coordination. Together, these effects, observed in the preventive and therapeutic treatments, demonstrate the efficiency of the EB101 vaccine in preventing and reducing amyloid burden and consequently, the resulting neurodegeneration. Proinflammatory and immune responses, assessed at the end of the prophylactic and therapeutic treatments, showed no significant inflammatory reaction in the brain of EB101-immunized mice, contrary to the previously reported in APP tg mouse studies [79] and A*ß*-vaccinated AD patients [21]. It is well known that Th1 T cells are activated by certain interleukins (IL-12), producing proinflammatory cytokines such as IFN-**γ**  and TNF-*a*, which are involved in the mechanism of autoimmune encephalitis [78], an activation pattern believed to have caused meningoencephalitis in 6% of vaccinated patients, together with moderate-to-high levels of CD45 immunoreactivity, which may lead to enhanced phagocytosis and degradation of brain fibrillar A*ß *load as reported elsewhere [18, 80]. However, Th2 T cells produce IL-4, 6, 10, and 13, most activated by our EB101 vaccine in APPswe/PS1dE9 transgenic mice and believed to help humoral immunity, essential for helping antibody production and suppression of autoimmune encephalitis. EB101 mice showed increased levels of proliferative and immunoreactive T cells, as well as Th 2 cytokines (IL-5, 6, 10, and 13) and reduced levels of A*ß*-reactive T cells, B cells, and GFAP aggregates. Together with the high levels of IgG antibody production, this indicates that EB101 does not induce autoimmune encephalitis or other significant signs of inflammation processes, as measured by proinflammatory cytokines [54] or by astrocytosis markers [81] in EB101immunized mice, in contrast to those observed in the EB102 and PBS mouse groups. Previous studies using A*ß *immunotherapy in APP-tg mice have reported different A*ß *antibody levels, depending on the mouse model used, immunization methodology, and type of adjuvant for the vaccine formulation. Adjuvants have a significant impact on the immune response elicited [82], thus we strategically planned a novel adjuvant-liposomal vaccine formulation markedly different from Freund's adjuvant [7, 16] or Quil-A [6] used in previous studies. Furthermore, the significant effect of EB101, observed in mice after the establishment of A*ß *deposition and subsequent neuropathological changes, indicates that EB101 vaccine has a great potential as a therapeutic agent to reverse AD-like brain pathology, an indispensable feature of an immunotherapeutic approach [19, 40, 52, 84]. Although the mechanism by which immunization schemes decrease AD-like neuropathology and prevent or reverse neurodegeneration and behavior deficits has not yet been elucidated, different metabolic aspects that are not exclusive could be responsible for plaque clearance, including traffic of A*ß *antibodies to the brain, antibody epitope specificity [77], transport of soluble A*ß *into the plasma with a subsequent antibody-mediated degradation (peripheral sink hypothesis) [41], and IgG transport systems [50]. The concept that providing increased specificity for the immune response decreases the probability of activating inflammatory response has been supported by many authors [42, 50] and appears to be confirmed by our present findings. However, we are aware that despite the marked reduction in astrocytosis and proinflammatory reaction elicited by EB101, as shown by the reduction of the specific markers examined, including the marked decrease of activated GFAP, B, and Timmunoreactive cells and Th2-elicited response, this effect warrants further preclinical and clinical investigations. Furthermore, in a clinical background, it is becoming clear that the therapeutic response to different forms of pharmacological intervention is genotype-specific [43, 46]. In this regard, a differential response to A*ß *immunization may be expected, depending upon the genomic profile of each patient. In the transgenic model used in the present immunization paradigm, our results unequivocally demonstrate that the EB101 vaccine may not only halt AD-related pathology and behavioral impairment but may actually reverse it, leading to a robust immunological response and an anti-inflammatory effect.

In summary, we have demonstrated that delivering A*ß *in a liposomal-S1P-adjuvant resulted in an effective immunological response with a wide spectrum of activities, including a reduction of the amyloid burden, reduction of NFTs, and induction of a Th2 T-cell response, which effectively prevented neuroinflammation in an animal model of AD. Our findings showed that EB101 immunization has a marked effect as a preventive and therapeutic treatment, not only in the major hallmarks of AD-like neuropathology but also in reducing amyloidosis-induced inflammation. Furthermore, we have shown that the liposomal/S1P adjuvant may act as an effective Th2 immunomodulator, stimulating innate immunity and enabling effective phagocytosis to clear amyloid and/or NFTs, thus reducing the risk of neuroinflammatory responses observed in other immunotherapeutic studies.

## 5. Conclusion

The main findings of this study show that (i) the AD-like hallmarks, beta amyloid plaques, and neurofibrillary tangle-like structures are strongly prevented or reduced under the different transgenic mouse treatments used in our experiments; (ii) activated astrocytes and neuroinflammation response, which spontaneously manifest as the AD pathology in APPswe/PS1dE9 transgenic mice, are reduced by treatment with our EB101 vaccine; (iii) a robust immune response was observed following our EB101 treatments, as determined by the A*β*-antibody titers detected in sera; (iv) the EB101 vaccine also resulted in an improvement in psychomotor activity as measured in the rota rod performance tests; (v) no negative effects on the brain cytoarchitecture, body motor-coordination, or behavioral patterns were observed in the EB101-treated mice. Studies to address whether the EB101 vaccine will be effective to inhibit AD-related pathology in other mouse models are needed before moving into clinical trials. 

## Figures and Tables

**Table 1:** Antibodies used for immunohistochemistry.

Antibody	Antigen	Type	Source	Dilut.	Reference
*β*-amyloid	Aβ_1-42 _ (mouse)	Mouse monoclonal	Millipore	1 : 1000	[40, 41]
Neurofibrillary tangle-like structure.	NFTs (rabbit)	Rabbit polyclonal	Millipore	1 : 300	[42, 43]
Glial fibrillary acidic p	GFAP (mouse)	Mouse monoclonal	Sigma	1 : 400	[14, 16, 44]
CD45RA	B-cells (mouse)	Mouse monoclonal	Dako	1 : 100	[15, 17, 45]
CD3	T-cells (rabbit)	Rabbit polyclonal	Dako	1 : 100	[17, 46]

## Abbreviations

AD: Alzheimer's disease
A*β*: *β*-amyloid protein
APP: amyloid precursor protein
APT: Anterior pretectal nucleus
CA1: Field CA1 hippocampus
CA3: Field CA3 hippocampus
Cpu: Caudate putamen (striatum)
Cx: Cortex
DG: Dentate gyrus
Ect: Ectorhinal cortex
GrDG: Granular dentate gyrus
IL: Interleukin
LPMR: Lateral posterior thalamic nucleus (mediorostral)
MODG: Molecular dentate gyrus
Pir: Piriform cortex
PoDG: Polymorph dentate gyrus
RSGb: Retrosplenial granular cortex b region
Tg: Transgenic
Th: T Helper.
